# Transarterial Infusion of Pembrolizumab Plus TACE for Liver Metastases of Melanoma

**DOI:** 10.1002/cnr2.70500

**Published:** 2026-02-17

**Authors:** Shahram Akhlaghpoor, Hamidreza Rouientan

**Affiliations:** ^1^ Department of Interventional Radiology Pardis Noor Medical Imaging and Cancer Center Tehran Iran

**Keywords:** hepatic arterial infusion, interventional oncology, melanoma, transarterial chemoembolization

## Abstract

**Background:**

Melanoma hepatic metastases demonstrate limited responsiveness to conventional systemic immune checkpoint blockade therapy, primarily attributed to the hepatic microenvironment's immunosuppressive characteristics. To our knowledge, this is the first reported case describing selective hepatic arterial infusion of pembrolizumab immediately followed by transarterial chemoembolization (TACE) for liver‐dominant melanoma metastases after progression on systemic therapy.

**Case Presentation:**

This case report describes an alternative therapeutic strategy employed for a 61‐year‐old female patient with hepatic metastases secondary to chemotherapy‐refractory uveal melanoma. Therapeutic intervention consisted of selective hepatic arterial pembrolizumab infusion immediately succeeded by TACE utilizing carboplatin, mitomycin, and idarubicin combined with lipiodol emulsion. Treatment cycles were administered at eight‐week intervals for two sessions. Radiological assessment 3 months post‐treatment demonstrated a complete response with absence of detectable hepatic malignancy.

**Conclusion:**

This clinical experience represents the initial documentation of sequential intraarterial pembrolizumab followed by TACE in chemotherapy‐resistant melanoma hepatic metastases. While a complete radiologic response was observed, conclusions regarding efficacy cannot be drawn from a single case. This approach appears feasible and warrants further study in well‐designed clinical investigations.

## 1 Introduction

Uveal melanoma is the most common primary intraocular malignancy in adults and is characterized by a strong hepatic tropism. Despite successful local treatment, approximately half of patients ultimately develop metastatic disease, and the liver is the first metastatic site in up to 90% of cases [1, 2]. Pembrolizumab is a standard therapy in advanced cutaneous melanoma, achieving objective response rates of 33% and durable long‐term survival [3, 4]. In contrast, systemic options are limited in metastatic uveal melanoma, and pooled outcomes with immune checkpoint inhibitors are low, with objective response rates around 3%–6% for single‐agent therapy [5]. Moreover, the efficacy of systemic pembrolizumab in patients with liver metastases has been suboptimal, with hepatic involvement often correlating with reduced therapeutic response to PD‐1 blockade therapy [6]. Therefore, liver‐directed modalities including transarterial chemoembolization (TACE), radioembolization, ablation, and hepatic perfusion are frequently considered for liver‐dominant metastatic uveal melanoma, although prospective data remain limited [7]. Experience with intra‐arterial immunotherapy in melanoma is extremely limited to date [8, 9]. Transarterial infusion of chemotherapy and immunotherapy allows delivery of agents directly into tumor‐feeding hepatic arteries and can achieve higher intratumoral drug exposure than systemic administration. In addition, TACE‐induced ischemic tumor injury can promote immunogenic cell death and antigen release, providing a biologic rationale for combination with immune checkpoint blockade [9, 10, 11, 12]. Given the limited efficacy of systemic immune checkpoint inhibitors in metastatic uveal melanoma, we report a liver‐directed strategy consisting of hepatic arterial pembrolizumab infusion followed immediately by TACE, aiming to maximize intrahepatic drug exposure and local tumor control.

## 2 Case Presentation

**FIGURE 1:**
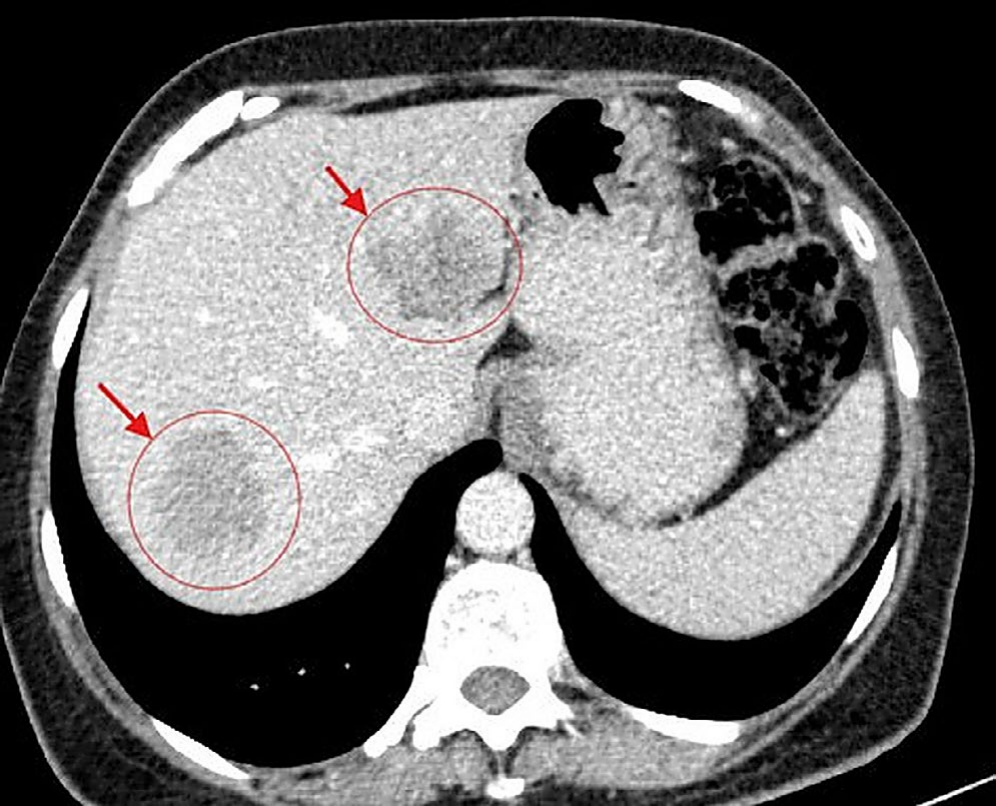
Baseline axial contrast‐enhanced computed tomography of the abdomen demonstrates hypodense lesions (red arrows) consistent with metastatic melanoma deposits involving both hepatic lobes.

**FIGURE 2:**
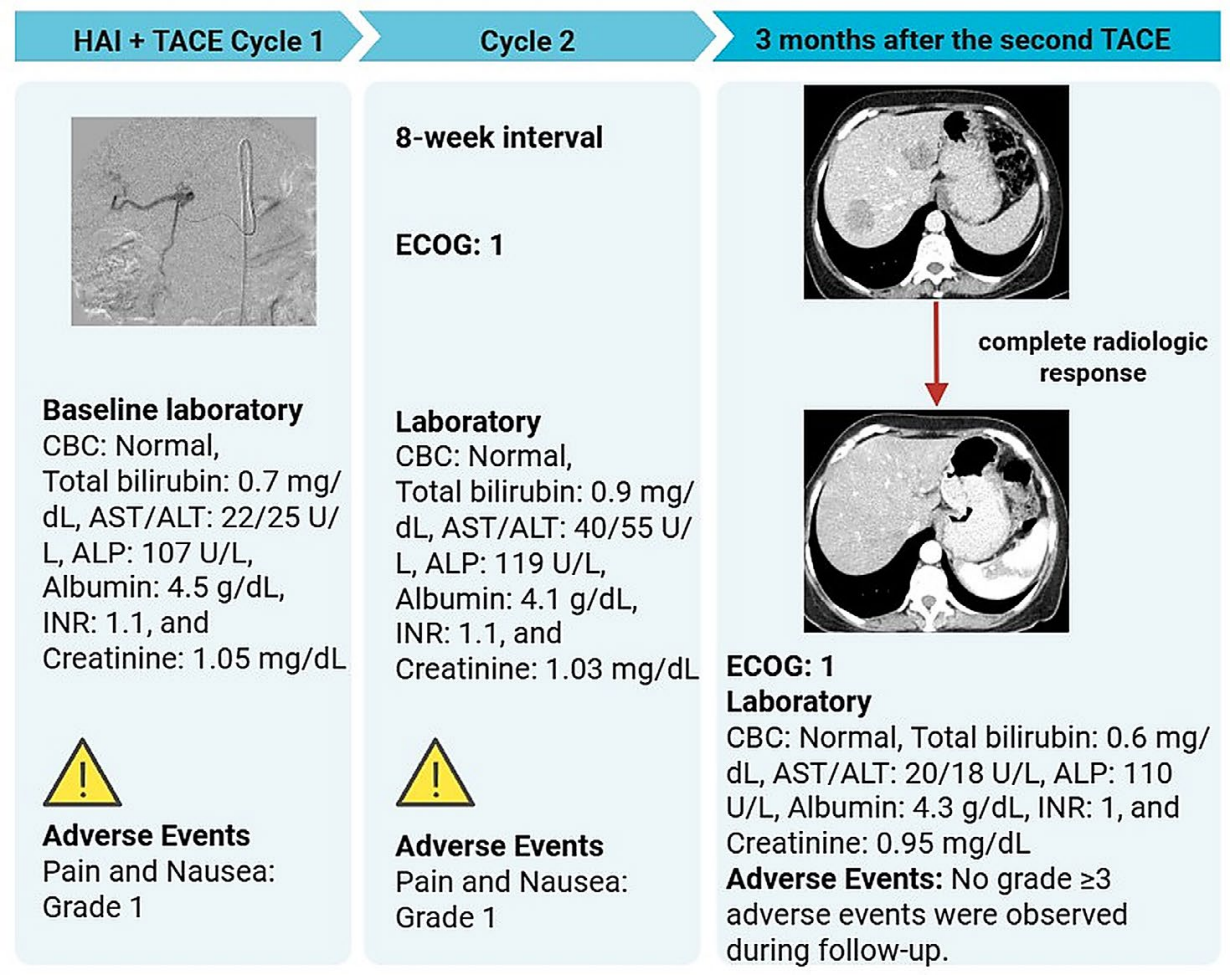
Clinical timeline of sequential HAI pembrolizumab and TACE (2 cycles, at 8‐week interval) with ECOG status, laboratory monitoring, and adverse‐event reporting. Follow‐up CT at 3 months after the second TACE shows a complete radiologic response. Adverse events were CTCAE‐graded; grade 1 pain and nausea occurred, and no grade ≥ 3 events were observed.

**FIGURE 3:**
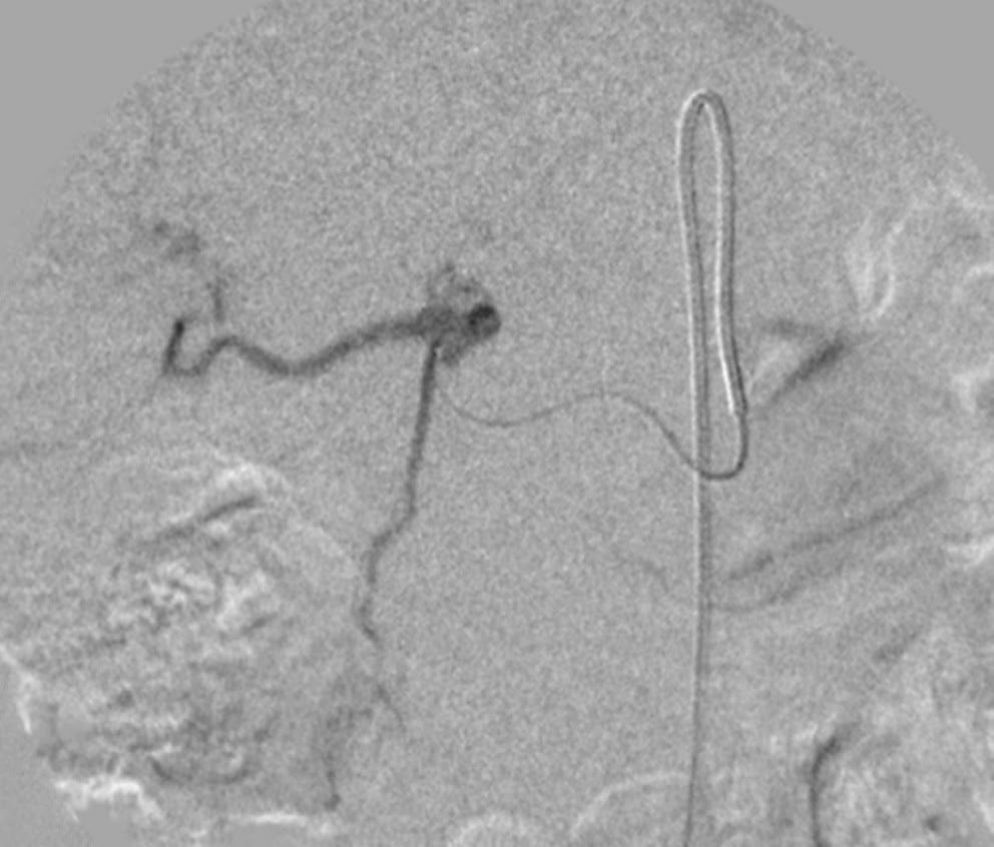
Digital subtraction angiographic image obtained during selective catheterization of the common hepatic artery utilizing a 5‐French diagnostic catheter via transfemoral approach.

**FIGURE 4:**
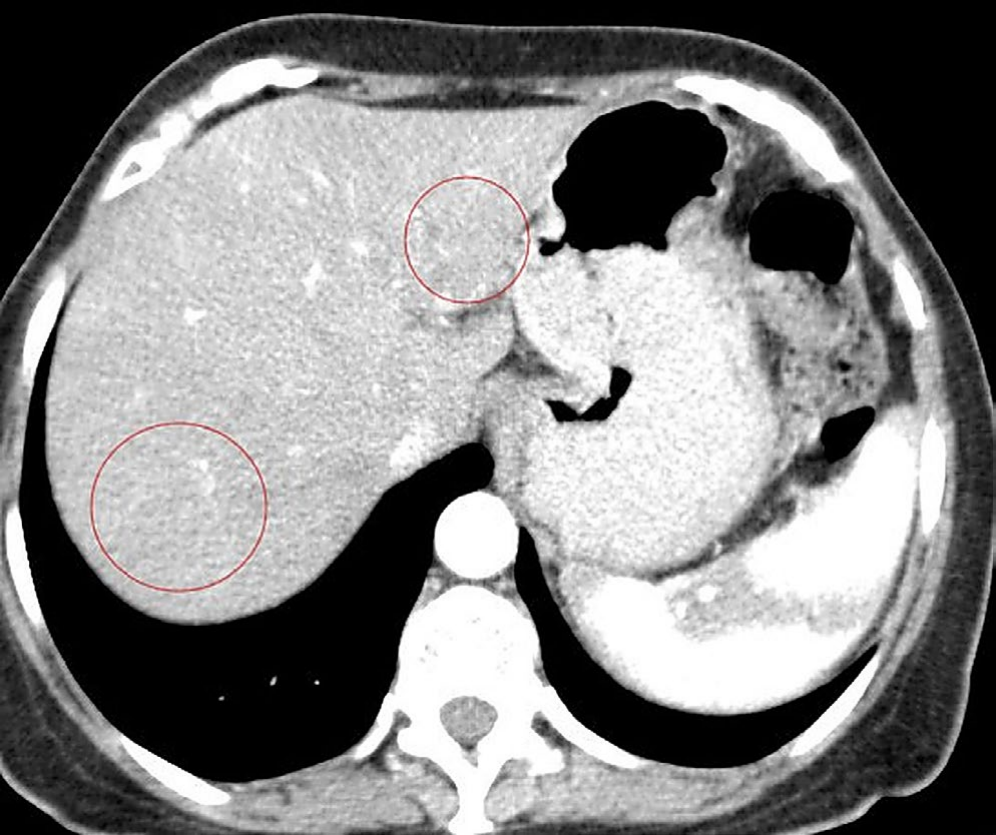
Follow‐up axial contrast‐enhanced computed tomography of the abdomen obtained 3 months following completion of the second treatment cycle demonstrates complete radiological response. No residual enhancing lesions are identified within either hepatic lobe (red circles denote the prior lesion locations).

In February 2024, a 61‐year‐old woman with a history of primary uveal melanoma developed hepatic metastases during routine surveillance imaging at Pardis Noor Medical Imaging and Cancer Center Complex. The patient was initiated on systemic chemotherapy protocols (Cisplatin 80 mg/m^2^ [on day 1], Dacarbazine 250 mg/m^2^ [on day 1–3], Vinblastine 2 mg [on day 1], administered every 3 weeks). Despite undergoing multiple cycles of conventional systemic chemotherapy plus systemic pembrolizumab over a 6‐month period, serial imaging demonstrated progressive disease. As shown in Figure 1, baseline imaging with contrast enhanced computed tomography (CT) of the abdomen demonstrated hepatic lesions involving both lobes of the liver. Given the lack of response to conventional systemic therapy and the patient's preserved performance status (Eastern Cooperative Oncology Group [ECOG] performance status of 1), a multidisciplinary interventional oncology consultation was obtained. Baseline and interval laboratory investigations (CBC, total bilirubin, AST/ALT, ALP, albumin, INR, and creatinine) were obtained before each cycle and during follow‐up and are summarized in the timeline (Figure 2). After comprehensive review of treatment options and informed consent discussion regarding the experimental nature of the proposed therapy, the patient was enrolled for combined hepatic arterial infusion immunotherapy and TACE. Under conscious sedation and local anesthesia, transfemoral arterial access was obtained using the Seldinger technique. Selective catheterization of the common hepatic artery was performed under digital subtraction angiography using a 5‐French catheter (Figure 3). Pembrolizumab 100 mg was diluted in 20 mL of 0.9% sodium chloride solution and administered via selective hepatic arterial infusion over 10 min at a controlled infusion rate of 2 mL/min. Immediately following pembrolizumab infusion, TACE was performed. A mixture of chemotherapeutic agents (Carboplatin 50 mg, Mitomycin 10 mg, and Idarubicin 10 mg) emulsified with iodized oil (Lipiodol Ultra‐Fluid; Laboratoire Andre Guerbet, Aulnay sous‐Bois, France) was injected through the microcatheter (Merit Maestro, Merit Medical Systems, Utah, USA) into the arterial branches feeding the tumor‐bearing segments of the liver. The endpoint of the TACE was near stasis of arterial flow in the treated territory, and the patient was monitored in the recovery unit for 6 h post procedure and discharged home without complications. This combined treatment approach was repeated at 8‐week intervals for a total of two treatment cycles. No procedure‐related adverse events occurred per SIR classification criteria [13]. Post‐embolization symptoms including pain and nausea were present and graded as CTCAE grade 1. On follow‐up, 3 months after the second TACE, a contrast enhanced CT scan of the abdomen was obtained. Follow‐up imaging demonstrated a complete radiological response with no evidence of residual hepatic metastases (Figure 4).

## 3 Discussion

Several liver‐directed modalities are used for metastatic uveal melanoma. These include percutaneous ablation, chemoembolization, radioembolization, and hepatic perfusion approaches. Most published evidence for these techniques is based on heterogeneous cohorts and prospective comparative data remain limited, making cross‐modality comparisons challenging [7, 14]. We report a patient with liver‐dominant metastatic uveal melanoma progressing after systemic therapy who underwent selective hepatic arterial pembrolizumab infusion immediately followed by TACE. The most significant finding was an early radiologic complete response on short follow‐up, supporting the technical feasibility of this sequencing approach. The major challenges are that safety data for regional anti‐PD‐1 delivery combined with embolization are limited, and in a single case, the relative contribution of arterial pembrolizumab versus TACE cannot be determined. Therefore, this report is hypothesis‐generating rather than evidence of efficacy. In addition, TACE itself can cause post‐embolization syndrome and less commonly clinically significant complications such as hepatic insufficiency/failure, biliary injury, liver abscess, cholecystitis, and non‐target embolization [15]. Importantly, liver enzyme elevations after combined therapy may be multifactorial (ischemic/embolization injury vs. immune‐mediated hepatitis), underscoring the need for protocolized monitoring and standardized attribution in future studies.

The theoretical foundation for intra‐arterial pembrolizumab administration is based on several pharmacokinetic and immunological principles. The dual blood supply of the liver allows for selective targeting of metastatic lesions, which derive their vasculature predominantly from the hepatic artery, while sparing normal hepatic parenchyma supplied by the portal circulation [16]. This anatomical advantage enables delivery of higher local drug concentrations directly to tumor sites while minimizing systemic exposure and associated toxicities [17, 18]. Likewise, the liver represents a uniquely immunosuppressive microenvironment that may limit the efficacy of systemically administered checkpoint inhibitors [19, 20]. These data support investigation of strategies that intensify local therapy within the liver. TACE of hepatic metastases induces hypoxia and tissue necrosis, which triggers the release of damage‐associated molecular patterns and exposes tumor‐associated antigens along with neoantigens [12, 21]. Clinical and translational studies in hepatocellular carcinoma suggest TACE can modulate immune pathways and have motivated combination strategies with PD‐1/PD‐L1 blockade [12, 22, 23]. Although these data are not directly generalizable to metastatic uveal melanoma, they support the clinical premise that TACE‐immunotherapy combinations can be delivered with manageable safety under protocolized monitoring. Locoregional immune stimulation has been explored in metastatic uveal melanoma using transarterial immunoembolization with GM‐CSF. In a double‐blind randomized phase II study, immunoembolization induced more robust post‐procedural inflammatory cytokine responses (including early IL‐6 and delayed IL‐8 signals) compared with bland embolization, and cytokine changes correlated with time to extrahepatic progression [24]. These findings support the plausibility of immune modulation after arterial embolotherapy. However, no immune biomarkers were measured in this case. Therefore, any immune mechanism remains speculative. Additionally, the lipiodol‐chemotherapy emulsion may also function as a sustained‐release depot, providing prolonged local exposure to cytotoxic agents.

Our approach builds upon the limited existing literature on intra‐arterial pembrolizumab administration. Chen et al. reported successful treatment of four patients with anorectal malignant melanoma using transcatheter arterial infusion of pembrolizumab (100 mg every 3 weeks) combined with systemic chemotherapy, achieving a pathological complete response in one patient and partial responses in two others without Grade 3–4 toxicities [8]. Similarly, The CATAP trial established the viability of combining cryoablation with hepatic arterial pembrolizumab delivery for treating melanoma hepatic metastases, demonstrating a 26.7% overall response rate [9]. Unlike these reports, our approach used immediate conventional lipiodol‐based TACE after arterial pembrolizumab infusion.

In conclusion, this case illustrates the technical feasibility of delivering pembrolizumab via hepatic arterial infusion immediately followed by TACE for liver‐dominant metastatic melanoma after progression on systemic therapy. The main lesson is that tightly sequenced regional immunotherapy–embolization strategies may be considered as hypothesis‐generating options in carefully selected patients within multidisciplinary decision‐making. Durable benefit, optimal patient selection, and safety require confirmation in prospective studies.

## Author Contributions

All of the authors had substantial contributions to the conception and interpretation of the work, drafting the work and revising it critically for important intellectual content. All the authors agreed on the final version to be published.

## Funding

The authors have nothing to report.

## Ethics Statement

The ethics of this study have been approved by the Ethical committee of Pardis Noor Medical Imaging and Cancer Center with the following code: PNMICC‐1404‐1008. All procedures performed in studies involving human participants were in accordance with the ethical standards of the institutional and/or national research committee and with the 1964 Helsinki declaration and its later amendments or comparable ethical standards.

## Consent

Written informed consent was obtained from the patient prior to the procedure, publication of the case details and accompanying images.

## Conflicts of Interest

The authors declare no conflicts of interest.

## Data Availability

The data that support the findings of this study are available on request from the corresponding author. The data are not publicly available due to privacy or ethical restrictions.
